# First wave COVID-19 pandemic in Senegal: Epidemiological and clinical characteristics

**DOI:** 10.1371/journal.pone.0274783

**Published:** 2022-09-20

**Authors:** Maryam Diarra, Aliou Barry, Ndongo Dia, Mamadou Diop, Ibrahima Sonko, Samba Sagne, Fatoumata Diene Sarr, Cheikh Talla, Adama Tall, Joseph Faye, Boly Diop, Cheikh Tidiane Diagne, Aboubacry Gaye, Amadou Diallo, Rose Mbaye, Mamadou Cisse, Fabien Taieb, Oumar Faye, Ndeye Aissatou Lakhe, Ba Papa Samba, Khardiata Diallo, Ndeye Maguette Fall, Aboubakar Sadikh Badiane, Louise Fortes, Moustapha Diop, Daouda Thioub, Alioune Badara Ly, Ousmane Faye, Moussa Seydi, Abdoulaye Bousso, Amadou A. Sall, Cheikh Loucoubar, Senegalese District Medical Officers

**Affiliations:** 1 Epidemiology, Clinical Research and Data Science Department, Institut Pasteur de Dakar, Dakar, Senegal; 2 Virology Department, Institut Pasteur de Dakar, Dakar, Senegal; 3 Centre des Opérations d’Urgence Sanitaire (COUS), Ministry of Health, Dakar, Senegal; 4 Division Surveillance Epidémiologique, Direction de la Prévention, Ministry of Health, Dakar, Senegal; 5 Diatropix Unit, Institut Pasteur de Dakar, Dakar, Senegal; 6 Service des Maladies Infectieuses, Fann Hospital, Dakar, Senegal; 7 Hospital Militar Principal, Dakar, Senegal; 8 Hospital Dalal Jam, Dakar, Senegal; Health Directorate, LUXEMBOURG

## Abstract

**Background:**

The novel coronavirus disease 2019 (COVID-19) pandemic has spread from China to the rest of the world. Africa seems less impacted with lower number of cases and deaths than other continents. Senegal recorded its first case on March 2, 2020. We present here data collected from March 2 to October 31, 2020 in Senegal.

**Methods:**

Socio-demographic, epidemiological, clinical and virological information were collected on suspected cases. To determine factors associated with diagnosed infection, symptomatic disease and death, multivariable binary logistic regression and log binomial models were used. Epidemiological parameters such as the reproduction number and growth rate were estimated.

**Results:**

67,608 suspected cases were tested by the IPD laboratories (13,031 positive and 54,577 negative). All age categories were associated with SARS-CoV-2 infection, but also patients having diabetes or hypertension or other cardiovascular diseases. With diagnosed infection, patients over 65 years and those with hypertension and cardiovascular disease and diabetes were highly associated with death. Patients with co-morbidities were associated with symptomatic disease, but only the under 15 years were not associated with. Among infected, 27.67% were asymptomatic (40.9% when contacts were systematically tested; 12.11% when only symptomatic or high-risk contacts were tested). Less than 15 years-old were mostly asymptomatic (63.2%). Dakar accounted for 81.4% of confirmed cases. The estimated mean serial interval was 5.57 (± 5.14) days. The average reproduction number was estimated at 1.161 (95%CI: 1.159–1.162), the growth rate was 0.031 (95%CI: 0.028–0.034) per day.

**Conclusions:**

Our findings indicated that factors associated with symptomatic COVID-19 and death are advanced age (over 65 years-old) and comorbidities such as diabetes and hypertension and cardiovascular disease.

## Introduction

Since December 31, 2019, the world has been affected by an unprecedented Coronavirus disease (COVID-19) pandemic. As of January 30, 2020, the World Health Organization (WHO) declared the pandemic a public health emergency of international concern [1]. Transmission of COVID-19 is known to mostly occur through direct contact with infected individuals or contaminated objects [2]. COVID-19 can also be spread by close contact with symptomatic patients via airborne microdroplets [3]. For more than 2 years, the COVID-19 pandemic has been ongoing and quickly spreading worldwide. As of February 15, 2022, and based on the WHO COVID-19 Dashboard, 410,565,868 confirmed cases and 5,810,880 deaths, with a global death-to-case ratio of 1.41% were reported worldwide [4]. Compared to the rest of the world, Africa remains the continent least affected with 11,041,815 confirmed cases and 244,440 deaths (death-to-case ratio of 2.21%) [5].

Among African countries, South Africa is the most affected with 3,641,811 confirmed cases and 96,993 deaths [5]. Senegal reported its first case on March 2, 2020; the 4 first cases recorded were imported from France and Italy [6]. Since then, the epidemic continues spreading across the country. As of February 15, 2022, the Senegalese Ministry of Health reported 85,412 confirmed cases and 1,957 deaths, with a death-to-case ratio of 2.29% [7]. In response to the COVID-19 pandemic, the Senegalese government implemented several measures since the first 14 confirmed cases. Chronologically, public gatherings including religious and cultural events were banned, Universities and schools were closed, international and commercial flights were stopped, inter-city transport was restricted, a state of health emergency was declared, and wearing a mask became mandatory. From March 2 to October 31, 2020, the Senegalese Ministry of Health adopted two different testing strategies. For the first period (from March 2 to the end of June 2020), all suspected cases and their contacts were systematically tested. For the second period (from the end of June to October 31, 2020), only suspected cases and their contacts with symptoms or co-morbidities were tested.

To better understand the epidemiological characteristics and the dynamics of the COVID-19 pandemic in Senegal, this study reports the results of a descriptive analysis from March 2 to October 31, 2020 of the pandemic surveillance.

## Materials and methods

### Field investigation method

This is a prospective case-ascertained study of COVID-19 suspected cases and their contacts between March 2 and October 31, 2020. Suspected cases were identified through an alert system set up by the Ministry of Health or from physicians at health care centers according to the Senegalese surveillance protocol. SARS-CoV-2 positive patients were notified by the laboratory to their home health district, which took charge of their medical follow-up. Medical care was administered at treatment centers or patients’ domicile depending on the severity of the infection.

### Case definitions and inclusion criteria

We used WHO case definitions [8] for suspected, confirmed and contact cases.

### Data collection method

Data were obtained through face-to-face interviews of the case (or family members if the case was too ill to be interviewed). Information was recorded on standardized investigation forms with a case identifier and sent to Institut Pasteur de Dakar (IPD) with the biological sample for testing. Collected information were: socio-demographic (age, sex, occupation, location), epidemiological (sampling date, geographical district, date of onset of symptoms, potential sources of infection (exposure), travel history, number of close contacts), clinical (body temperature, symptoms including cough, sore throat, headache, arthralgia, myalgia, nasal discharge, nasal congestion, anosmia, ageusia, difficulty of breathing, nausea/vomiting, diarrhea) and presence of comorbidity (Diabetes, asthma, hypertension and cardiovascular diseases). Field investigation forms were then checked before data entry and storage into an electronic central MySQL database. In this study, the deaths recorded are patients who were diagnosed at the Pasteur Institute de Dakar, hospitalized in health centres and whose death was documented by the Ministry of Health. For hospitalized patients, we defined the duration of SARS-CoV-2 carriage as the time in days between the first and last positive SARS-CoV-2 PCR test. Delay to consultation is defined as the time in days between the date of symptoms onset and date of first positive SARS-CoV-2 PCR test.

### Laboratory tests techniques

Oro and/or nasopharyngeal swabs specimens were collected from suspected patients or persons in contact with confirmed cases and placed in a universal viral transport medium, stored at 4–8°C and transported to IPD within 24 hours of collection for testing. Once in the laboratory, specimens were immediately processed for SAR-SCoV-2-specific real-time RT-PCR for viral nucleic acid detection. At least two aliquots of each sample were also stored at −80°C for biobanking or additional analysis.

### Disease outcomes (patients’ status)

Patients were classified according to "uninfected/infected" status and "asymptomatic/symptomatic" as well as "survival/dead" status among infected. Patients’ data were analyzed in detail and risk factor analysis was performed to identify determinants of infection, asymptomatic infection and death.

### Statistical analysis

The epidemic curve was established based on daily incidence. We evaluated potential risk factors for COVID-19 illness, including sex, age group ([0–15], [15–45], [45–65], [65–100] years-old), period of testing strategy, occupation (public transportation drivers, heath care workers, students and/or teachers, traders as well as other non-listed occupations) and presence of comorbidity (Diabetes, hypertension and cardiovascular diseases, asthma). The normality test for age distribution was performed using Anderson-Darling test with the R software “nortest” package [9]. For non-normal data, a non-parametric Mann Whitney U test was used to compare the average age between the first and second period of testing strategy. The Fisher exact test was used to compare the categorical variables between the two periods. In this study we have two types of data: i) a surveillance data (a sample of people undergoing diagnostic testing–and this has a mix of symptomatic individuals being investigated, and contacts of diagnosed cases) and ii) a cohort data (a follow-up of hospitalised patients to assess their outcome). For "uninfected/infected" and "asymptomatic/symptomatic" status from surveillance data, statistical analysis were performed using univariate binary logistic regression models. Crude results from univariate models are provided in forest plots and tables. Significant variables from the univariate analysis were included to the multivariable binary logistic regression analysis to assess a statistically significant association between the independent variables and the response variable using Adjusted Odd Ratio (AOR), 95% CIs for AOR and p-values. A backward procedure was applied to select the variables that remained significant with a p-value < 0.05 in the final model. For the cohort data, the same statistical analysis procedure was applied as before using a log binomial model on the response variable "survival/dead". Adjusted Relative Risk (ARR), 95% CIs for ARR and p-values were used for testing significance and interpretation of results.

We estimated the serial interval from groups of infected individuals in chains of transmission for whom information on the date of symptom onset was available. Based on the estimates of the serial interval, we calculated the reproduction number (R_0_), which is the average number of secondary cases that arise when one primary case is introduced into the population. If the value of R_0_ is greater than 1, the infection may spread in the population. The time required for the incidence to double, namely the doubling time was computed based on the parameters of a log-linear model fitted to the growth phase of the epidemic. R_*t*_, the instantaneous effective reproduction number, represents the expected number of secondary cases arising from a primary case infected at time *t*. It is an important parameter to assess the effectiveness of public health interventions [10,11]. R_*t*_ was estimated according to incidence data and serial interval parameters using the R software EpiEstim package [12].

Statistical analysis and mapping were performed using the R4.0.1 statistical language environment [13].

### Ethics statement

As part of the national COVID-19 pandemic surveillance in Senegal, there is no need for approval by the National Ethics Committee of the Ministry of Health. Data were collected in an objective of surveillance and are anonymous. Consent was informed for their data to be used for surveillance and/or research purposes. Verbal consent was obtained from the patient prior to taking an oro/nasopharyngeal swab sample. Throughout the surveillance, the generated database in Institut Pasteur de Dakar was shared with the Senegalese Ministry of Health and Social Action.

## Results

### Descriptive analysis

We present here Senegalese COVID-19 epidemic data collected from March 2 to October 31, 2020. The first positive case was confirmed on March 2, 2020, and was a traveler from France. We analyzed data related to patients tested in the IPD laboratories, which were the main national laboratory covering up to 80% of total samples collected from the whole country. There were 67,608 COVID-19 suspected individuals investigated with 13,031 (19.27%) confirmed cases among them.

**Fig 1:**
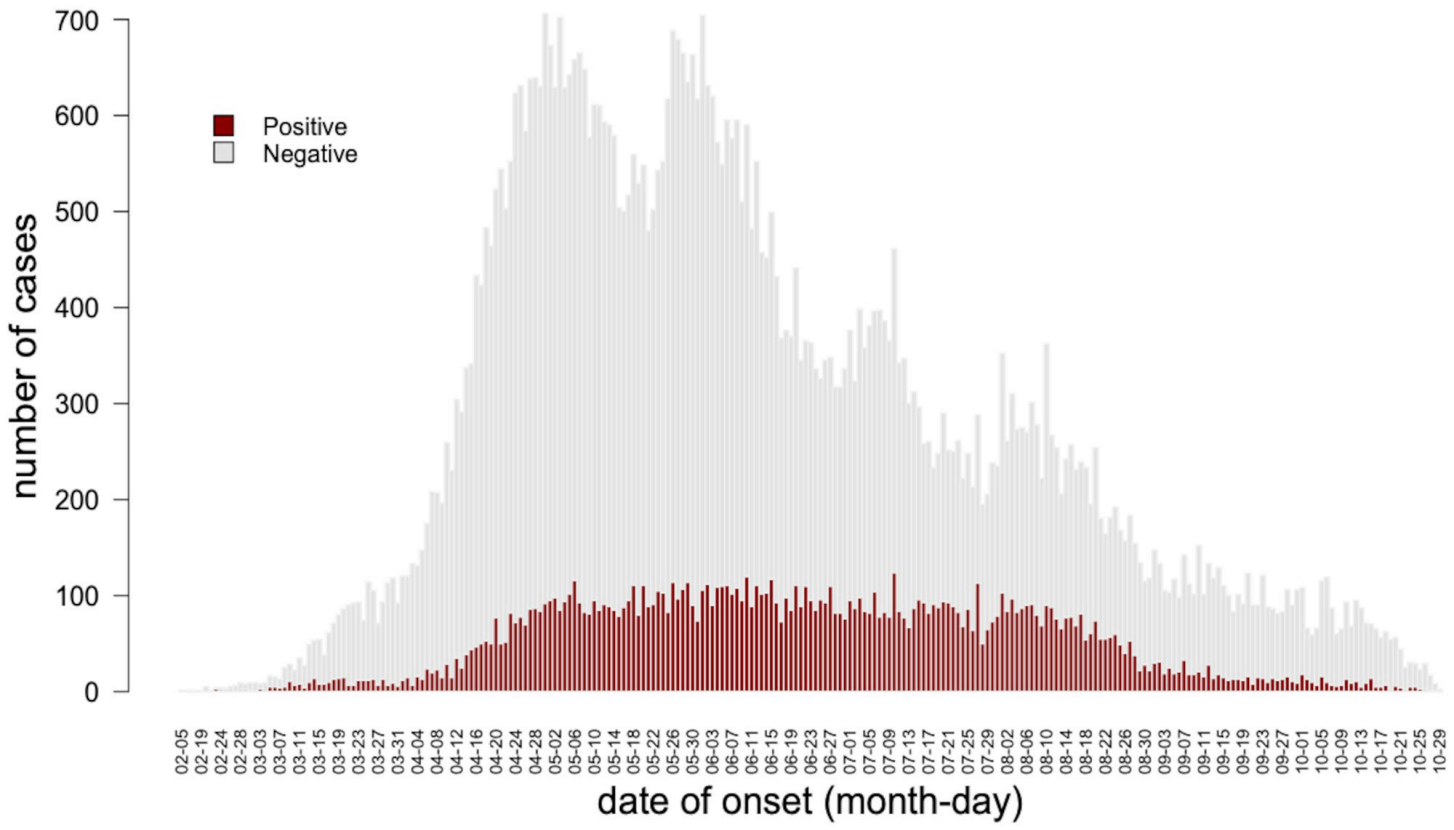
Epidemic curve. Dates of onset are represented in the x-axis and number of cases on the y-axis. The top of the vertical bars indicates the total number of new suspected cases per day. Grey part of the bars represents negative cases while red part positive cases.

During these first 8 months of the COVID-19 outbreak in Senegal, the epidemic curve showed a low incidence at the beginning, a considerable increase by mid-April and a decrease from mid-August until October 31, 2020. The number of new cases per day ranged from 0 to 32 (mean = 9.2(± 8.1)) from the beginning of the outbreak to April 14, between 37 and 134 (mean = 88.5(±15.4)) from April 15 to August 14 and from 89 to 0 (mean = 22.2(±20.7)) from August 15 to October 31 (Fig 1).

**Fig 2:**
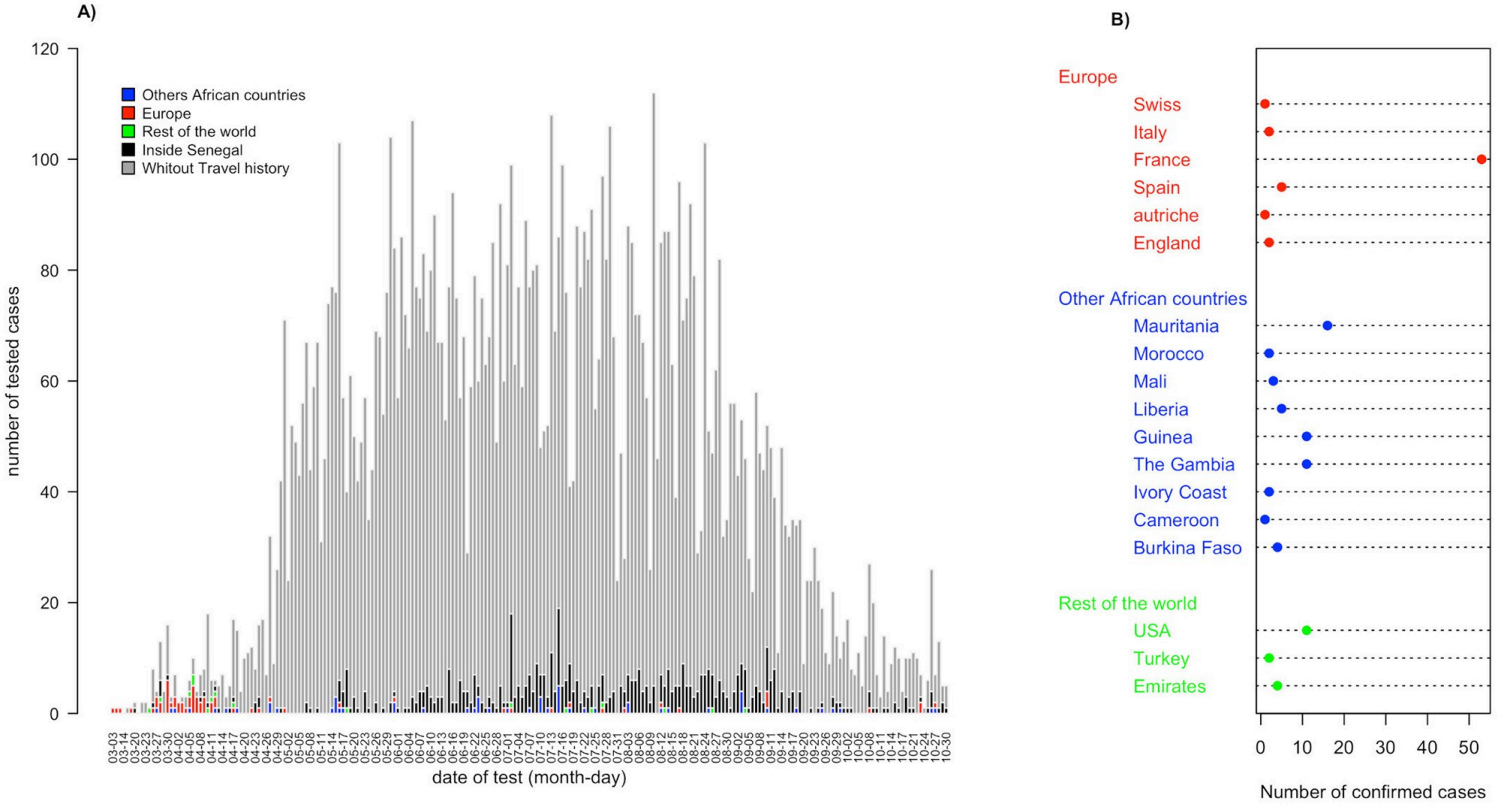
Confirmed COVID-19 cases according to their travel history. A. Histogram of COVID-19 cases with and without travel history. Dates of onset are represented in the x-axis and number of tested cases on the y-axis. Grey part of the bars represents cases without travel history, black bars cases circulated inside Senegal, green bars cases from the rest of the world, red bars cases from Europe and blue bars cases from other African countries. B. Country’s origin of imported COVID-19 cases.

Among these 13,031 confirmed cases, 1,352 (10.38%) had an international travel history within 14 days preceding the onset of illness. Fig 2A shows that the proportion of confirmed cases having an international travel history was higher at the beginning of the epidemic and were mostly from Europe, France in particular. For the overall study period, the most frequent sources of the imported infections are France (53 cases), Mauritania (16), USA (11), The Gambia (11) and Guinea (11) (Fig 2B). Other countries have less than 10 imported cases (Fig 2B). For the reported period, one index case had an average of 12.6 contacts (min-max: 1–313) and an average of 2 secondary cases (min-max: 0–52) arise among these contacts.

Overall, the male/female sex ratio in suspected cases was 1.21 but was similar in confirmed (1.22) and negative cases (1.20), suggesting an absence of gender effect on the infection risk. The mean age of infected patients was 41.2 (±19.5) years. Among the most reported occupations (public transportation drivers, healthcare workers, students and teachers, traders), traders were the most affected (24.3%) category while students and teachers the least (17.5%) (Table 1).

**Table 1:** Univariate and multivariable factors analysis according to "uninfected/infected" status.

Variables	Labels	Number of suspected cases (%)	Number of positive cases	Proportion of positive cases (%)	Univariate Logistic Regression			Multivariable Logistic Regression		
					Crude OR	95CI	p-value	Adjusted OR	95CI	Adjusted p-value
**Sex**	Female	30264 (44.8)	5847	19.3	1	-	-			
	Male	36513 (54)	7110	19.5	1.01	(0.97–1.05)	< 0.001			
	Missing Sex	831 (1.2)	74	8.9						
**Age groups**	[0–15]	7878 (11.7)	935	11.9	1	-	-	1	-	-
	[15–45]	38293 (56.6)	6861	17.9	1.62	(1.51–1.74)	< 0.001	1.18	(1.03–1.36)	0.01
	[45–65]	14220 (21)	3233	22.7	2.19	(2.02–2.36)	< 0.001	1.59	(1.38–1.85)	< 0.001
	[65–100]	6286 (9.3)	1915	30.5	3.25	(2.98–3.55)	< 0.001	2.06	(1.75–2.41)	< 0.001
	Missing Age	931 (1.4)	87	9.3						
**Period of testing strategy**	First_period	44154 (65.3)	7044	16	-	-	-	-	-	-
	Second_period	23454 (34.7)	5987	25.5	1.81	(1.74–1.88)	< 0.001	1.08	(1.02–1.14)	0.007
**Occupation**	Others	26761 (39.6)	6094	22.8	-	-	-	-	-	-
	Drivers	1037 (1.5)	183	17.6	0.73	(0.62–0.85)	< 0.001	0.87	(0.71–1.06)	0.134
	Medical_staff	3982 (5.9)	719	18.1	0.75	(0.69–0.81)	< 0.001	0.87	(0.78–0.96)	0.004
	Students_teachers	6663 (9.9)	1164	17.5	0.72	(0.67–0.77)	< 0.001	0.98	(0.9–1.08)	0.627
	Traders	3072 (4.5)	747	24.3	1.09	(1.00–1.19)	0.054	1.23	(1.11–1.37)	< 0.001
	Missing Occupation	26093 (38.6)	4124	15.8						
**Diabetes**	No	37741 (55.8)	10020	26.5	1	-	-			
	Yes	753 (1.1)	288	38.2	1.71	(1.48–1.99)	< 0.001			
	Missing Diabetes	29114 (43.1)	2723	9.4						
**Hypertension Cardiovascular disease**	No	38189 (56.5)	10143	26.6	1	-	-	1	-	-
	Yes	312 (0.5)	172	55.1	3.4	(2.71–4.25)	< 0.001	2.34	(1.75–3.12)	< 0.001
	Missing HCD	29107 (43.1)	2716	9.3						
**Asthma**	No	37992 (56.2)	10176	26.8	-	-	-			
	Yes	486 (0.7)	116	23.9	0.86	(0.69–1.06)	0.149			
	Missing Asthma	29130 (43.1)	2739	9.4						

OR = Odds Ratio; 95CI = 95% Confidence Interval.

HCD = Hypertension Cardiovascular disease.

Among the 13,031 positives cases, there were 3,606 asymptomatic infections (27.7%). Among infected children (less than 15 years old), 63.2% were asymptomatic. (Table 2).

**Table 2:** Univariate and multivariable factors analysis according to "symptomatic/asymptomatic" status.

Variables	Labels	Number of positive cases (%)	Number of Symptomatic cases	Proportion of Symptomatic (%)	Univariate Logistic Regression			Multivariate Logistic Regression		
					Crude OR	95CI	p-value	Adjusted OR	95CI	Adjusted p-value
**Sex**	Female	5847 (44.87)	4237	72.5	-	-	-			
	Male	7110 (54.56)	5136	72.2	1.01	(0.93–1.1)	0.814			
	Missing Sex	74 (0.57)	52	70.3						
**Age groups**	[0–15]	935 (7.18)	344	36.8	-	-	-	-	-	-
	[15–45]	6861 (52.65)	4840	70.5	4.28	(3.7–4.96)	< 0.001	4.21	(3.5–5.06)	< 0.001
	[45–65]	3233 (24.81)	2587	80	7.28	(6.18–8.57)	< 0.001	6.07	(4.96–7.44)	< 0.001
	[65–100]	1915 (14.7)	1616	84.4	10.13	(8.38–12.25)	< 0.001	6.77	(5.38–8.52)	< 0.001
	Missing Age	87 (0.67)	38	43.7						
**Period of testing strategy**	First_period	7044 (54.06)	4163	59.1	-	-	-			
	Second_period	5987 (45.94)	5262	87.9	5.53	(5.01–6.1)	< 0.001	4.19	(3.75–4.68)	< 0.001
**Diabetes**	No	10020 (76.89)	7810	77.9	-	-	-			
	Yes	288 (2.21)	255	88.5	2.41	(1.64–3.52)	< 0.001			
	Missing Diabetes	2723 (20.9)	1360	49.9						
**Hypertension Cardiovascular disease**	No	10143 (77.84)	7915	78	-	-	-	-	-	
	Yes	172 (1.32)	158	91.9	3.71	(2.06–6.67)	< 0.001	3.26	(1.67–6.34)	< 0.001
	Missing HCD	2716 (20.84)	1352	49.8						
**Asthma**	No	10176 (78.09)	7953	78.2	-	-	-			
	Yes	116 (0.89)	101	87.1	1.88	(1.09–3.24)	0.023			
	Missing Asthma	2739 (21.02)	1371	50.1						

OR = Odds Ratio; 95CI = 95% Confidence Interval.

HCD = Hypertension Cardiovascular disease.

**Fig 3:**
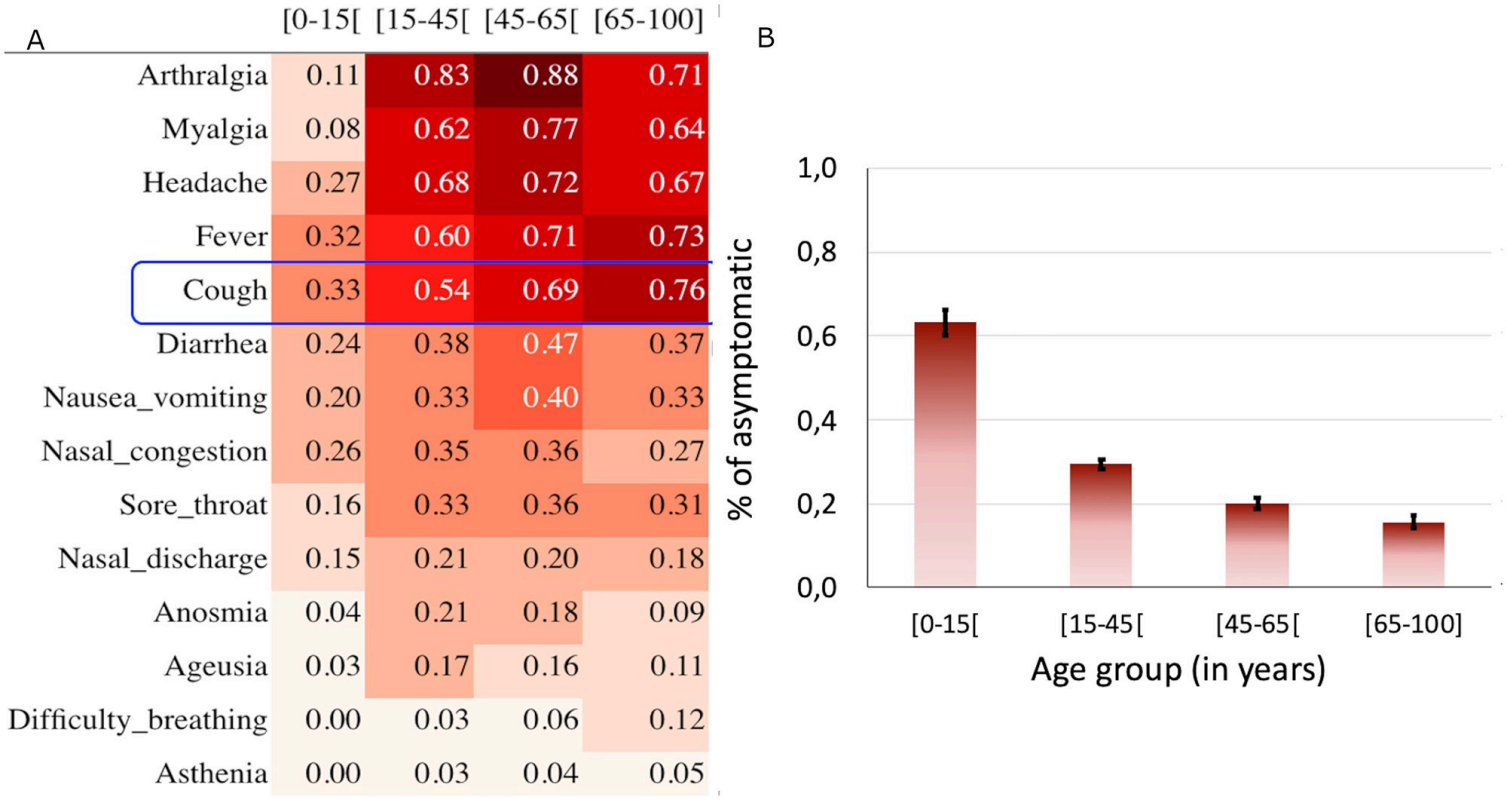
Heatmap of symptom frequency and histogram of asymptomatic patients by age group. A. Heatmap of symptom frequency by age group. Symptoms are listed in rows and age groups in columns. Values in cells indicate the frequency of patients from the corresponding age group manifesting the corresponding symptom. The more the red color is accentuated, the more the symptom is frequent. The blue box targets the "cough" symptom, one of the most involved symptoms in transmission due to the dispersed micro-droplets. This symptom is less frequent in young patients (most active sub-population) compared to adult (less active sub-population). B. Histogram of asymptomatic patients by age group. Black vertical bars represent the standard errors.

From symptomatic infections, the most common symptoms reported were: headache (66.4%), fever (63.5%), cough (59.9%), sore throat (32.7%) and nasal discharge (20.3%), anosmia (17.4%) and ageusia (15.4%) (Fig 3A). The frequency of fever, cough and sore throat was higher in patients over 45 years compared to others (Fig 3B).

### Spatial distribution of COVID-19 cases through the country

**Fig 4:**
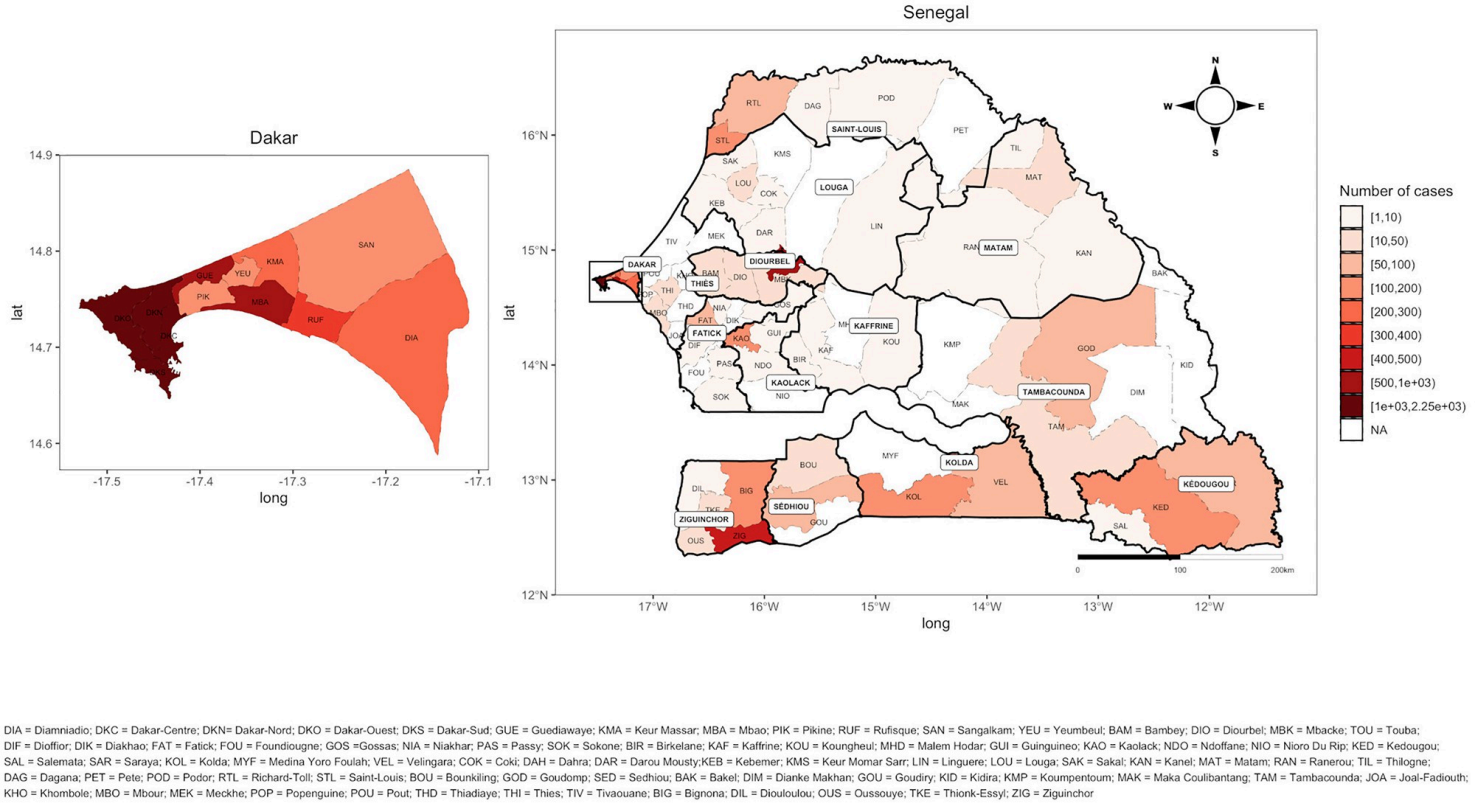
Spatial distribution of COVID-19 positive cases at district level.

The spatial distribution of confirmed cases (Fig 4) showed that all 14 regions in Senegal were affected chronologically (Dakar, Diourbel, Thies, Saint-Louis, Ziguinchor, Fatick, Kolda, Tambacounda, Louga, Kaolack, Sedhiou, Kedougou, Kaffrine and Matam). On March 2, 2020, the first case occurred in Dakar Ouest health district, in Dakar region. On March 11th, Touba health district (in Diourbel region) reported its first case. During March, the epidemic expanded to the neighboring districts of Dakar and spread to all other regions in Senegal. Up to October 31, 2020, Dakar remained the most affected region by COVID-19 accounting for 81.4% of confirmed infections. The cumulative number of confirmed cases for each region shows that Dakar was the epicenter of the epidemic followed by Diourbel region (S1 Fig). 61 health districts (out of 79) were affected (Table 4). The most affected districts were all from Dakar region, one from Diourbel region (the district of Touba) and those situated on the border with other countries (Saint-Louis and Richard-Toll in the North, Matam and Goudiry in the East and almost all health districts in the South) (Fig 4). The highest attack rates observed concerned several health districts in Dakar (Dakar Sud, Dakar Centre, Dakar Ouest, Dakar Nord, Guediawaye, Diamniadio, Mbao and Sangalkam), Ziguinchor and Kedougou districts (S6 Table).

### Multivariable risk factors analysis for "uninfected/infected" status

**Fig 5:**
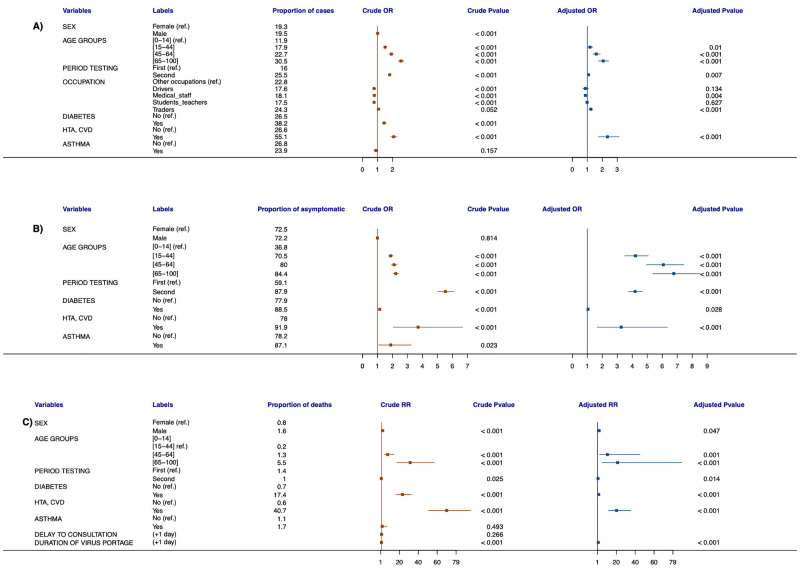
Forest plot for COVID-19 associated factors. A. Forest plot for factors associated with COVID-19 infection. B. Forest plot for factors associated with COVID-19 symptomatic disease. C. Forest plot for factors associated with COVID-19 mortality. Vertical lines (red and blue) represent odds ratio of 1. Red (respectively blue) dots represent crude (respectively adjusted) odds ratio. Red (respectively blue) horizontal lines around dots represent 95% confidence intervals for crude (respectively adjusted) odds ratio.

Table 1 provides information on comorbidities and socio-demographic characteristics from suspected and confirmed COVID-19 cases and shows that gender, age group and some occupations are significant factors associated with SARS-CoV-2 infection. For the whole study period, compared to the under 15 years, the odds for being infected by COVID-19 were more likely among patients other 15 years old (AOR = 1.18, 95%CI = 1.03–1.36, p-value = 0.01 for 15–44 years; AOR = 1.59, 95%CI = 1.38–1.85, p-value < 0.001 for 45–64 years and AOR = 2.06, 95%CI = 1.75–2.41, p-value < 0.001 for over 65 years) (Table 1). Regarding the period of testing strategy, the odds for being infected during the second period was 1.08 times than in the first period (AOR = 1.08, 95%CI = 1.02–1.14, p-value < 0.007). Factors such as occupation showed significant associations with COVID-19 infection risk (Fig 5A). The odds for being infected among traders was 1.23 times than other occupation (AOR = 1.23, 95%CI = 1.11–1.37, p-value < 0.001). Compared to all other occupations, health care workers were weakly associated with infection (AOR = 0.87; 95%CI 0.78–0.96; p-value = 0.004). The odds for being infected among patients with hypertension and cardiovascular disease was 2.34 times compared to patients with other declared comorbidities (AOR = 2.34, 95%CI = 1.75–3.12, p-value < 0.001).

Factors found associated with SARS-CoV-2 infection during the first period (from March 2 to June 25, 2020) and second period (from June 26 to October 31, 2020) are similar for those on the entire study period. Regarding age group, the odds are higher in the second period (AOR = 1.84 for 15–44 years, AOR = 2.52 for 45–64 years, AOR = 2.78 for >65 years (S2 Table)) than in the first period (AOR = 1.04 for 15–44 years, AOR = 1.35 for 45–64 years, AOR = 2.28 for >65 years (S1 Table)). However, regarding comorbidities, the odds for being infected was higher in the first period (AOR = 3.2 (S1 Table)) than the second period (AOR = 1.89 (S2 Table)).

### Multivariable risk factors analysis for "symptomatic/asymptomatic" status

On the entire study period, the proportions of symptomatic cases increase with age (Fig 5B): 36.8% for patients less than 15 years old, 70.5% for 15–44 years, 80% for 45–64 years and 84.4% for patients more than 65 years. The odds of developing symptomatic disease were more likely among patients over 15 years (AOR = 4.21, 95%CI = 3.35–5.06, p-value < 0.001 for 15–44 years; AOR = 6.07, 95%CI = 4.96–7.44, p-value < 0.001 for 45–64 years and AOR = 6.77, 95%CI = 5.38–8.52, p-value < 0.001 for ≥65 years). During the second period of testing strategy, patients were 4.19 times more likely to be symptomatic than in the first period (AOR = 4.19, 95%CI = 3.75–4.68, p-value < 0.001). Furthermore, regarding comorbidities, the odds of developing symptomatic COVID-19 was 3.26 times among patients with hypertension and cardiovascular disease compared to patients with no such illness (AOR = 3.26, 95%CI = 1.67–6.34, p-value < 0.001) (Table 2).

In the first study period, the factors associated with symptomatic COVID-19 are similar to the whole study period (S3 Table). There were 22.5% of asymptomatic children. However, the odds of developing symptomatic disease were lower in the second period (AOR = 2.14, 95%CI = 1.38–3.31, p-value < 0.001 for 15–44 years; AOR = 2.35, 95%CI = 1.49–3.7, p-value < 0.001 for 45–64 years and AOR = 1.99, 95%CI = 1.25–3.18, p-value = 0.004 for ≥65 years) (S4 Table).

### Multivariable risk factors analysis for "survival/dead" status

Fig 5C shows that age is an associated factor for death. Table 3 shows that sex, age groups, some comorbidities and the duration of coronavirus carriage were significantly associated with mortality in confirmed infections. Coronavirus mortality was 1.45 times higher in men than women (p-value = 0.046). There was no mortality recorded for patients less than 15 years old. Among declared comorbidities, the relative risk for COVID-19 mortality is higher for patients with hypertension and cardiovascular disease (ARR = 20.23, 95%CI = 11.68–35.04, p < 0.001), followed by patients having diabetes (ARR = 1.31, 95%CI = 0.77–2.23, p-value < 0.001). The average duration of coronavirus carriage in infected patients was 4.93(± 5.77) days and 2.4 (± 5.77) days for infections leading to death.

**Table 3:** Univariate and multivariable factors analysis according to "survival/dead" status.

Variables	Labels	Number of positive cases (%)	Number of Deaths	Death-to-case ratio (%)	Univariate log binomial model			Multivariate log binomial model		
					Crude RR	95CI	p-value	Adjusted RR	95CI	Adjusted p-value
**Sex**	Female	5847 (44.9)	44	0.8	1	-	-	1	-	-
	Male	7110 (54.6)	114	1.6	2.13	(1.51–3.01)	< 0.001	1.45	(0.84–2.5)	0.046
	Missing Sex	74 (0.6)	1	1.4						
**Age groups**	[0–15]	935 (7.2)	0	0	-	-	-	-	-	-
	[15–45]	6861 (52.7)	12	0.2	1	-	-	1	-	-
	[45–65]	3233 (24.8)	42	1.3	7.43	(3.92–14.09)	< 0.001	10.56	(2.48–44.96)	0.001
	[65–100]	1915 (14.7)	105	5.5	31.35	(17.29–56.84)	< 0.001	21.23	(5.05–89.24)	< 0.001
	Missing Age	87 (0.7)	0	0						
**Period of testing strategy**	First_period	7044 (54.1)	100	1.4	-	-	-	1	-	-
	Second_period	5987 (45.9)	59	1	0.69	(0.5–0.96)	0.025	0.56	(0.35–0.89)	0.014
**Diabetes**	No	10020 (76.9)	75	0.7	1	-	-	1	-	-
	Yes	288 (2.2)	50	17.4	23.19	(16.54–32.53)	< 0.001	1.31	(0.77–2.23)	< 0.001
	Missing Diabetes	2723 (20.9)	34	1.2						
**Hypertension Cardiovascular disease**	No	10143 (77.9)	59	0.6	1	-	-	1	-	-
	Yes	172 (1.3)	70	40.7	69.97	(51.22–95.57)	< 0.001	20.23	(11.68–35.04)	< 0.001
	Missing HCD	2716 (20.8)	30	1.1						
**Asthma**	No	10176 (78.1)	108	1.1	1	-	-			
	Yes	116 (0.9)	2	1.7	1.62	(0.41–6.5)	0.493			
	Missing Asthma	2739 (21)	49	1.8						
**Delay to consultation**		mean(sd)	mean(sd) in deaths							
		4.59 (3.6)	5.7 (3.6)	-	1.02	(0.98–1.06)	0.266			
**Duration of virus portage**		4.93 (5.77)	2.4 (5.77)	-	0.89	(0.85–0.93)	< 0.001	0.88	(0.82–0.94)	< 0.001

RR = Relative Risk; 95CI = 95% Confidence Interval.

HCD = Hypertension Cardiovascular disease.

### Modelling

**Fig 6:**
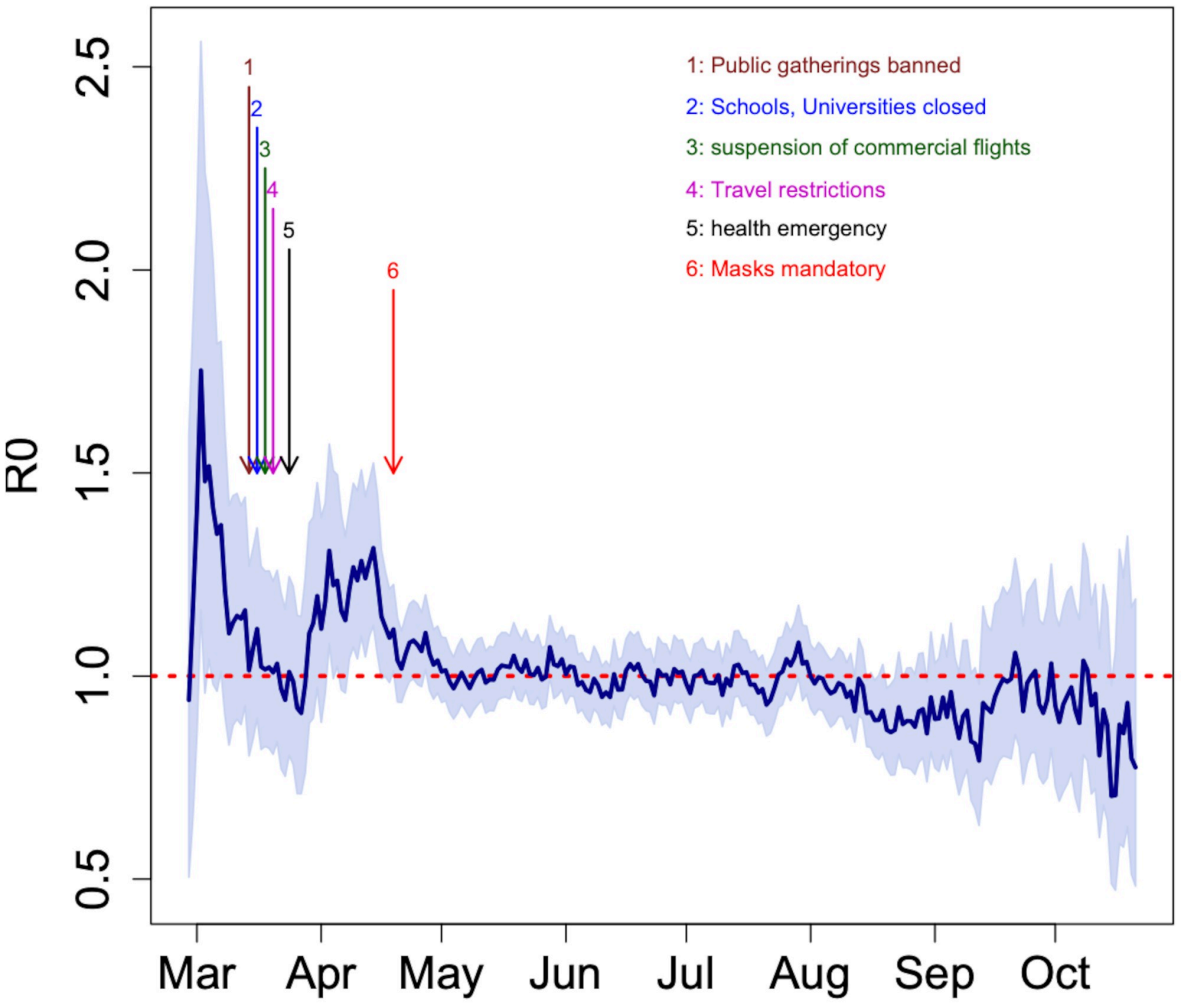
Evolution of the reproduction number across time. Y-axis gives the estimated reproduction number and X-axis the dates. The curve in solid line shows the dynamic of reproduction number. The shaded region around the curve represents the 95% confidence interval. Vertical arrows indicate official dates of the intervention measures taken the Senegalese government.

The estimated mean serial interval for COVID-19 infection in Senegal was 5.57 (± 5.14) days (S2 Fig). The reproduction number over time (R_*t*_) was estimated for each day of the outbreak. During the course of the 8 months of the epidemic, R_0_ started with the maximum value of 1.75 (95%CI 1.16–2.56). R_0_ decreased until the end of March with the five series of measures (banning public gathering, closing schools and universities, suspending commercial flights, travel restrictions and health emergency), then increased again before decreasing after mid-April with obligatory mask-wearing. (Fig 6). The estimated basic reproduction number over the study period was 1.161 (95%CI = 1.159–1.162). During this period, the epidemiological parameters of the epidemic in Senegal were similar to those in Dakar region. A slow growth rate (0.031 per day, 95%CI = 0.028–0.034) of the epidemic in Senegal was observed, with a similar rate for Dakar (0.033 per day, 95%CI = 0.03–0.037). As of October 31, 2020, the doubling time of the epidemic in Senegal was 22 days (95%CI = 20–25 days), 20 days (19–23) in Dakar (S5 Table).

The comparison of epidemiological parameters according to the two periods (before and after changing testing strategies) is presented in Table 4. The age distribution in the population was significantly different from normal distribution (Anderson-Darling test p-value < 0.001). The population was relatively younger in period 1 with average age = 33.3 (±18.1) years-old compared to period 2 with average age = 42.5 (±19.1) years-old (Wilcoxon test p-value <0.001). There was significantly more man than women in period 1 (M/F sex ratio = 1.26) compared to period 2 (M/F sex ratio = 1.1), Fisher-Exact test p-value < 0.001. However, the proportion of SARS-CoV-2 infected patients are higher in the second period (25.5%) than in the first (16%), Fisher-Exact test p-value < 0.001. Among the SARS- CoV-2 infected patients, there were globally 27.7% asymptomatic patients: 41% in the first period and 12.1% in the second period. The reproduction number on the overall period is similar to the one on the first period. For the second period, the reproduction number was around 1 corresponding to the moment when the epidemic was stable.

**Table 4:** Epidemiological parameters comparison according to different periods.

Epidemiological parameters	Period 1	Period 2	Global
	March–June 25, 2020	June 26 –October 31, 2020	March–October 31, 2020
Number of suspected cases tested	44154	23454	67608
Number of Sars-Cov-2 confirmed cases (%)	7044 (16%)	5987 (25.53%)	13031 (19.27%)
Mean age in years (min—max)	33.3 (0–100)	42.5 (0–100)	36.5 (0–100)
Sex ratio (male/female)	1.26	1.1	1.21
% of asymptomatic among all tested individuals	70.43	19.05	52.6
% of asymptomatic among Sars-Cov-2 confirmed individuals	40.9	12.11	27.67
Estimated Reproduction Number (95CI)	1.17 (1.16–1.18)	1.038 (1.036–1.040)	1.161 (1.159–1.162)
Average number of contacts tested per index	16	6	12
Average number of secondary cases per index	2.4	1.9	2
Number of sanitary districts affected	53	58	61

## Discussion

This study gives important insight into the pandemic in West Africa as there has been a relative paucity of clinical and epidemiological data from this region. In addition to the study conducted by Lawson and colleagues [14] in Thiès region (1 out of the 14 regions) in Senegal, this study reinforces the socio-demographic and epidemiological information on COVID-19 by extending to all other regions in the country. During the first weeks of the epidemic, almost all confirmed cases were imported from Europe, specially from France. Among the symptomatic infections, the most important signs and symptoms associated with COVID-19 reported in this study (headache, fever, cough, sore throat and nasal discharge among others) were similar to those reported elsewhere [15,16]. The index case giving the most secondary cases had 167 contacts and infected 52 of them. This super-spreader was a religious teacher with about several hundred disciples in his school (as named "daarah") located in the southern region of the country.

The proportion of infected health care workers is relatively low due to higher awareness of set protective measures. In contrast, this study shows that traders are at high risk of being infected by SARS-CoV-2. Closing shops was not part of the protective measures taken by the government. Given that the shops are usually hubs, traders were most exposed as they receive considerable flux of customers from unknown sources daily.

Regarding age groups, they are all susceptible to having SARS-CoV-2 infection, particularly older people were more associated to infection. Furthermore, after adjustment on sex, age group and occupation, patients having hypertension and cardiovascular disease are more highly associated with SARS-CoV-2 infection. Among infected, the two youngest age groups represent 60% of this study cohort and were mostly asymptomatic. They are known to be generally more active than the remaining age groups with more mobility. They coughed less and had fewer sore throats and fevers than older people. Coughing is one of the symptoms contributing most to the spread of the virus due to the projected microdroplets. This could explain why the transmission of the virus is relatively slow in Senegal.

Our results show that men are twice more likely to die from COVID-19, but also advanced age (over 65 years old) and the presence of comorbidities such as diabetes and hypertension and cardiovascular diseases are associated factors for COVID-19 death. This is was not surprising and is consistent with what has been observed not only in Africa [14,17–22] but in other countries of the world [23–26]. Indeed, studies conducted in Spain by Pastor-Barriuso and colleagues [25] showed that older men are more exposed to coronavirus mortality. Pantea Stoian and colleagues [24] confirmed also that males, diabetes and hypertension are associated factors for coronavirus mortality.

The highest SARS-CoV-2 infection’s cases noted in Dakar (the capital of Senegal) may be related to its high population size and high inter-urban mobility as reported by the national agency of statistic and demography of Senegal (http://www.ansd.sn/). This is concordant to the trend of the other countries where COVID-19 epicenters were noticed into their largest cities as showed in world meters’ data [27]. This study, based on surveillance data, confirms the trends in the spatial distribution of COVID-19 observed cases in the national seroprevalence survey. Indeed, the most affected regions described in our study were confirmed by the seroprevalence survey [22] with the exception of the Thies region. In fact, the IPD laboratory had data from the Thies region for only the 2 first months of the pandemic. Compared to the seroprevalence data, our study demonstrated that the surveillance data is only a small part of the iceberg and does not really reveal the real number of COVID-19 infected people during the first wave.

The trend of our estimates on the instantaneous reproduction number R_*t*_ may suggest that the first couple of control measures that the Senegalese government has taken were effective for about the 2 first weeks slowing down this parameter below 1. However, the rise of the R_*t*_ noticed after the 2 first weeks, could suggest that those measures were no longer effective or there has been laxity on restriction’s measures upon population. As of April 19, 2020, the government imposed wearing masks. Since then, R_*t*_ decreased and remains below 1 until October 31, 2020.

Our estimates of serial interval and reproduction number are similar to other reports [28,29]. The mean reproduction number found in Senegal was relatively small (1.138 (1.136–1.139)) and the epidemic trajectory slow compared to many others countries. As of April 11, 2020, studies conducted in Nigeria [30] estimated a reproduction number of 1.42 (95%CI 1.26–1.58). However, these reproduction numbers found in Africa are very low compared to those noted elsewhere. Indeed, reproduction number was 2.2 (95%CI 1.4–3.9) in China [3,31], 3.27 (95%CI 3.17–3.38) for Italy, 6.32 (95%CI 5.72–6.99) for France, 6.07 (95%CI 5.51–6.69) for Germany, and 5.08 (95%CI 4.51–5.74) for Spain with an exponential growth method [32] and 5.30 ± 0.95 in the United States of America [33]. This low reproduction number found in Senegal could be explained by the early implementation of public health interventions from the first case detection. Indeed, African countries have been very early warned of the risks of COVID-19 importations [34].

## Conclusions

During the eight months of COVID-19 pandemic in Senegal, the virus spread all around the 14 regions but the incidence curve is very flat. We found a slow growth rate and noticed that the number of cases doubled in size approximately every 22 days. Imposing mask-wearing seems to be one of the most effective control measures. Our findings indicated that older men, diabetes and hypertension are associated factors for coronavirus mortality and provide important parameters for further analyses such as the evaluation of the impact of control measures, and the chains of transmission on COVID-19 disease in Senegal. This work is also an important pre-requisite for future analysis about the particularities of each wave through a comparative study of the different waves of COVID-19 in Senegal.

## Supporting information

S1 Fig Cumulative incidence of COVID19 in Senegal by region. (DOCX)

S2 Fig Distribution of serial interval. (DOCX)

S1 Table Univariate and multivariate risk factors analysis according to "uninfected/infected" status for Period 1 (from March 2 to June 25,2020). (DOCX)

S2 Table Univariate and multivariate risk factors analysis according to "uninfected/infected" status for Period 2 (from June 26 to October 31^st^,2020). (DOCX)

S3 Table Univariate and multivariate risk factors analysis according to "symptomatic/asymptomatic" status for Period 1 (from March 2 to June 25,2020). (DOCX)

S4 Table Univariate and multivariate risk factors analysis according to "symptomatic/asymptomatic" status for Period 2 (from June 26 to October 31^st^, 2020). (DOCX)

S5 Table Estimates of epidemiological parameters of COVID-19 outbreak in Senegal and the 3 most affected regions. (DOCX)

S6 Table COVID-19 Attack rate per 100,000 inhabitants (hbts) by gender and health district. (DOCX)

S1 File (DOCX)

S2 File (ZIP)
