# Supplementary material for: First wave COVID-19 pandemic in Senegal: Epidemiological and clinical characteristics
Source: PLoS One. 2022 Sep 20;17(9):e0274783. doi: 10.1371/journal.pone.0274783 (PMC9488827; doi:10.1371/journal.pone.0274783)
Supplement: S2 Fig — (DOCX) [file pone.0274783.s002.docx]

**Figure S2.** Distribution of serial interval
