# Supplementary material for: First wave COVID-19 pandemic in Senegal: Epidemiological and clinical characteristics
Source: PLoS One. 2022 Sep 20;17(9):e0274783. doi: 10.1371/journal.pone.0274783 (PMC9488827; doi:10.1371/journal.pone.0274783)
Supplement: S1 Table — (DOCX) [file pone.0274783.s003.docx]

**Table S1:** Univariate and multivariate risk factors analysis according to "uninfected/infected" status for Period 1 (from March 2 to June 25,2020)

|  | |  | |  | |  | |  | | | **Univariate log binomial model** | | | **Multivariable log binomial model** | | | |
| --- | --- | --- | --- | --- | --- | --- | --- | --- | --- | --- | --- | --- | --- | --- | --- | --- | --- |
| **Variables** | **Labels** | | **Number of suspected cases (%)** | | **Number of positive cases** | | **Proportion of positive cases (%)** | | **Crude RR** | **95CI** | | **p-value** | **Adjusted RR** | | **95CI** | **Adjusted p-value** |  |
| **Sex** | Female | | 10908 (46.5) | | 2747 | | 25.2 | | - | - | | - |  | |  |  |  |
|  | Male | | 12050 (51.4) | | 3187 | | 26.4 | | 1 | [0.96 ; 1.05] | | 0.953 |  | |  |  |  |
|  | Missing Sex | | 496 (2.1) | | 53 | | 10.7 | |  |  | |  |  | |  |  |  |
| **Age groups** | [0-15[ | | 1178 (5) | | 195 | | 16.6 | | - | - | | - | 1 | | - | - |  |
|  | [15-45[ | | 12272 (52.3) | | 2827 | | 23 | | 1.4 | [1.3; 1.51] | | < 0.001 | 1.03 | | [0.92 ; 1.16] | 0.63 |  |
|  | [45-65[ | | 6142 (26.2) | | 1748 | | 28.5 | | 1.66 | [1.53; 1.81] | | < 0.001 | 1.24 | | [1.09 ; 1.41] | < 0.001 |  |
|  | [65-100] | | 3616 (15.4) | | 1179 | | 32.6 | | 2.5 | [2.28; 2.74] | | < 0.001 | 1.7 | | [1.49 ; 1.95] | < 0.001 |  |
|  | Missing Age | | 246 (1) | | 38 | | 15.4 | |  |  | |  |  | |  |  |  |
| **Occupation** | Others | | 10257 (43.7) | | 2890 | | 28.2 | | - | - | | - | - | | - | - |  |
|  | Drivers | | 251 (1.1) | | 65 | | 25.9 | | 0.77 | [0.65; 0.92] | | 0.003 | 0.88 | | [0.73 ; 1.06] | 0.169 |  |
|  | Medical_staff | | 1276 (5.4) | | 364 | | 28.5 | | 0.68 | [0.61; 0.75] | | < 0.001 | 0.74 | | [0.66 ; 0.83] | < 0.001 |  |
|  | Students_teachers | | 1474 (6.3) | | 353 | | 23.9 | | 0.81 | [0.75; 0.86] | | < 0.001 | 0.97 | | [0.89 ; 1.06] | 0.492 |  |
|  | Traders | | 800 (3.4) | | 253 | | 31.6 | | 1.12 | [1.03; 1.22] | | 0.008 | 1.19 | | [1.09 ; 1.3] | < 0.001 |  |
|  | Missing Occupation | | 9396 (40.1) | | 2062 | | 21.9 | |  |  | |  |  | |  |  |  |
| **Diabetes** | No | | 17127 (73) | | 5027 | | 29.4 | | - | - | | - |  | |  |  |  |
|  | Yes | | 501 (2.1) | | 180 | | 35.9 | | 1.77 | [1.53; 2.04] | | < 0.001 |  | |  |  |  |
|  | Missing Diabetes | | 5826 (24.8) | | 780 | | 13.4 | |  |  | |  |  | |  |  |  |
| **Hypertension Cardiovascular disease** | No | | 17443 (74.4) | | 5128 | | 29.4 | | - | - | | - | 1 | | - | - |  |
|  | Yes | | 190 (0.8) | | 84 | | 44.2 | | 2.98 | [2.67; 3.34] | | < 0.001 | 1.75 | | [1.49 ; 2.05] | < 0.001 |  |
|  | Missing HCD | | 5821 (24.8) | | 775 | | 13.3 | |  |  | |  |  | |  |  |  |
| **Asthma** | No | | 17294 (73.7) | | 5127 | | 29.6 | | - | - | | - |  | |  |  |  |
|  | Yes | | 331 (1.4) | | 77 | | 23.3 | | 1.03 | [0.79; 1.35] | | 0.824 |  | |  |  |  |
|  | Missing Asthma | | 5829 (24.9) | | 783 | | 13.4 | | - | - | | - |  | |  |  |  |
