# Supplementary material for: First wave COVID-19 pandemic in Senegal: Epidemiological and clinical characteristics
Source: PLoS One. 2022 Sep 20;17(9):e0274783. doi: 10.1371/journal.pone.0274783 (PMC9488827; doi:10.1371/journal.pone.0274783)
Supplement: S2 Table — (DOCX) [file pone.0274783.s004.docx]

**Table S2:** Univariate and multivariate risk factors analysis according to "uninfected/infected" status for Period 2 (from June 26 to October 31^st^,2020)

|  | |  | |  | |  | |  | | | **Univariate log binomial model** | | | **Multivariable log binomial model** | | | |
| --- | --- | --- | --- | --- | --- | --- | --- | --- | --- | --- | --- | --- | --- | --- | --- | --- | --- |
| **Variables** | **Labels** | | **Number of suspected cases (%)** | | **Number of positive cases** | | **Proportion of positive cases (%)** | | **Crude RR** | **95CI** | | **p-value** | **Adjusted RR** | | **95CI** | **Adjusted p-value** |  |
| **Sex** | Female | | 10908 (46.5) | | 2747 | | 25.2 | | - | - | | - |  | |  |  |  |
|  | Male | | 12050 (51.4) | | 3187 | | 26.4 | | 1.05 | [1.01 ; 1.1] | | 0.029 |  | |  |  |  |
|  | Missing Sex | | 496 (2.1) | | 53 | | 10.7 | |  |  | |  |  | |  |  |  |
| **Age groups** | [0-15[ | | 1178 (5) | | 195 | | 16.6 | | - | - | | - | 1 | | - | - |  |
|  | [15-45[ | | 12272 (52.3) | | 2827 | | 23 | | 1.39 | [1.22 ; 1.59] | | < 0.001 | 1.6 | | [1.25 ; 2.05] | < 0.001 |  |
|  | [45-65[ | | 6142 (26.2) | | 1748 | | 28.5 | | 1.72 | [1.5 ; 1.97] | | < 0.001 | 1.98 | | [1.54 ; 2.54] | < 0.001 |  |
|  | [65-100] | | 3616 (15.4) | | 1179 | | 32.6 | | 1.97 | [1.72 ; 2.26] | | < 0.001 | 2.11 | | [1.64 ; 2.72] | < 0.001 |  |
|  | Missing Age | | 246 (1) | | 38 | | 15.4 | |  |  | |  |  | |  |  |  |
| **Occupation** | Others | | 10257 (43.7) | | 2890 | | 28.2 | | - | - | | - | - | | - | - |  |
|  | Drivers | | 251 (1.1) | | 65 | | 25.9 | | 0.92 | [0.74 ; 1.14] | | 0.434 | 0.96 | | [0.77 ; 1.2] | 0.727 |  |
|  | Medical_staff | | 1276 (5.4) | | 364 | | 28.5 | | 1.01 | [0.92 ; 1.11] | | 0.793 | 1.11 | | [1.01 ; 1.22] | 0.033 |  |
|  | Students_teachers | | 1474 (6.3) | | 353 | | 23.9 | | 0.85 | [0.77 ; 0.94] | | < 0.001 | 1.01 | | [0.91 ; 1.12] | 0.83 |  |
|  | Traders | | 800 (3.4) | | 253 | | 31.6 | | 1.12 | [1.01 ; 1.25] | | 0.034 | 1.07 | | [0.96 ; 1.19] | 0.245 |  |
|  | Missing Occupation | | 9396 (40.1) | | 2062 | | 21.9 | |  |  | |  |  | |  |  |  |
| **Diabetes** | No | | 17127 (73) | | 5027 | | 29.4 | | - | - | | - |  | |  |  |  |
|  | Yes | | 501 (2.1) | | 180 | | 35.9 | | 1.22 | [1.09 ; 1.38] | | < 0.001 |  | |  |  |  |
|  | Missing Diabetes | | 5826 (24.8) | | 780 | | 13.4 | |  |  | |  |  | |  |  |  |
| **Hypertension Cardiovascular disease** | No | | 17443 (74.4) | | 5128 | | 29.4 | | - | - | | - | 1 | | - | - |  |
|  | Yes | | 190 (0.8) | | 84 | | 44.2 | | 1.5 | [1.28 ; 1.77] | | < 0.001 | 1.45 | | [1.21 ; 1.73] | < 0.001 |  |
|  | Missing HCD | | 5821 (24.8) | | 775 | | 13.3 | |  |  | |  |  | |  |  |  |
| **Asthma** | No | | 17294 (73.7) | | 5127 | | 29.6 | | - | - | | - |  | |  |  |  |
|  | Yes | | 331 (1.4) | | 77 | | 23.3 | | 0.78 | [0.64 ; 0.96] | | 0.016 |  | |  |  |  |
|  | Missing Asthma | | 5829 (24.9) | | 783 | | 13.4 | | - | - | | - |  | |  |  |  |
