# Supplementary material for: First wave COVID-19 pandemic in Senegal: Epidemiological and clinical characteristics
Source: PLoS One. 2022 Sep 20;17(9):e0274783. doi: 10.1371/journal.pone.0274783 (PMC9488827; doi:10.1371/journal.pone.0274783)
Supplement: S3 Table — (DOCX) [file pone.0274783.s005.docx]

**Table S3.** Univariate and multivariate risk factors analysis according to "symptomatic/asymptomatic" status for Period 1 (from March 2 to June 25,2020)

|  |  |  |  |  | **Univariate log binomial model** | | | **Multivariate log binomial model** | | |
| --- | --- | --- | --- | --- | --- | --- | --- | --- | --- | --- |
| **Variables** | **Labels** | **Number of positive cases (%)** | **Number of Symptomatic cases** | **Proportion of Symptomatic (%)** | **Crude RR** | **95CI** | **p-value** | **Adjusted RR** | **95CI** | **Adjusted p-value** |
| **Sex** | Female | 3100 (44.01) | 1798 | 58 | - | - | - |  |  |  |
|  | Male | 3923 (55.69) | 2358 | 60.1 | 1.04 | [1.01 ; 1.08] | 0.025 |  |  |  |
|  | Missing Sex | 21 (0.3) | 7 | 33.3 |  |  |  |  |  |  |
| **Age groups** | [0-15[ | 740 (10.51) | 191 | 25.8 | - | - | - | - | - | - |
|  | [15-45[ | 4034 (57.27) | 2351 | 58.3 | 2.22 | [1.96 ; 2.51] | < 0.001 | 2.34 | [2.02 ; 2.71] | < 0.001 |
|  | [45-65[ | 1485 (21.08) | 1040 | 70 | 2.63 | [2.32 ; 2.98] | < 0.001 | 2.69 | [2.32 ; 3.13] | < 0.001 |
|  | [65-100] | 736 (10.45) | 568 | 77.2 | 2.92 | [2.57 ; 3.31] | < 0.001 | 2.89 | [2.48 ; 3.36] | < 0.001 |
|  | Missing Age | 49 (0.7) | 13 | 26.5 |  |  |  |  |  |  |
| **Diabetes** | No | 4993 (70.88) | 3278 | 65.7 | - | - | - | - |  |  |
|  | Yes | 108 (1.53) | 94 | 87 | 1.35 | [1.26 ; 1.45] | < 0.001 | 1.09 | [1.01 ; 1.18] | 0.032 |
|  | Missing Diabetes | 1943 (27.58) | 791 | 40.7 |  |  |  |  |  |  |
| **Hypertension Cardiovascular disease** | No | 5015 (71.2) | 3298 | 65.8 | - | - | - | - | - |  |
|  | Yes | 88 (1.25) | 77 | 87.5 | 1.35 | [1.24 ; 1.46] | < 0.001 | 1.14 | [1.06 ; 1.24] | < 0.001 |
|  | Missing HCD | 1941 (27.56) | 788 | 40.6 |  |  |  |  |  |  |
| **Asthma** | No | 5049 (71.68) | 3332 | 66 | - | - | - |  | - |  |
|  | Yes | 39 (0.55) | 31 | 79.5 | 1.2 | [1.03 ; 1.41] | 0.023 | 1 | [0.96 ; 1.04] | 0.986 |
|  | Missing Asthma | 1956 (27.77) | 800 | 40.9 |  |  |  |  |  |  |
