# Supplementary material for: First wave COVID-19 pandemic in Senegal: Epidemiological and clinical characteristics
Source: PLoS One. 2022 Sep 20;17(9):e0274783. doi: 10.1371/journal.pone.0274783 (PMC9488827; doi:10.1371/journal.pone.0274783)
Supplement: S4 Table — (DOCX) [file pone.0274783.s006.docx]

**Table S4.** Univariate and multivariate risk factors analysis according to "symptomatic/asymptomatic" status for Period 2 (from June 26 to October 31^st^, 2020)

|  |  |  |  |  | **Univariate log binomial model** | | | **Multivariate log binomial model** | | |
| --- | --- | --- | --- | --- | --- | --- | --- | --- | --- | --- |
| **Variables** | **Labels** | **Number of positive cases (%)** | **Number of Symptomatic cases** | **Proportion of Symptomatic (%)** | **Crude RR** | **95CI** | **p-value** | **Adjusted RR** | **95CI** | **Adjusted p-value** |
| **Sex** | Female | 2747 (45.88) | 2439 | 88.8 | - | - | - |  |  |  |
|  | Male | 3187 (53.23) | 2778 | 87.2 | 0.98 | [0.97; 1.01] | 0.062 |  |  |  |
|  | Missing Sex | 53 (0.89) | 45 | 84.9 |  |  |  |  |  |  |
| **Age groups** | [0-15[ | 195 (3.26) | 153 | 78.5 | - | - | - | - | - | - |
|  | [15-45[ | 2827 (47.22) | 2489 | 88 | 1.12 | [1.03; 1.21] | < 0.001 | 1.11 | [1.03 ; 1.2] | 0.01 |
|  | [45-65[ | 1748 (29.2) | 1547 | 88.5 | 1.13 | [1.03; 1.31] | < 0.001 | 1.12 | [1.03 ; 1.21] | 0.005 |
|  | [65-100] | 1179 (19.69) | 1048 | 88.9 | 1.13 | [1.03; 1.32] | < 0.001 | 1.1 | [1.01 ; 1.19] | 0.024 |
|  | Missing Age | 38 (0.63) | 25 | 65.8 |  |  |  |  |  |  |
| **Diabetes** | No | 5027 (83.97) | 4532 | 90.2 | - | - | - |  |  |  |
|  | Yes | 180 (3.01) | 161 | 89.4 | 0.99 | [0.61; 1.6] | 0.927 |  |  |  |
|  | Missing Diabetes | 780 (13.03) | 569 | 72.9 |  |  |  |  |  |  |
| **Hypertension Cardiovascular disease** | No | 5128 (85.65) | 4617 | 90 | - | - | - | - | - |  |
|  | Yes | 84 (1.4) | 81 | 96.4 | 1.07 | [1.01; 1.1] | 0.036 | 1.09 | [1.06 ; 1.12] | < 0.001 |
|  | Missing HCD | 775 (12.94) | 564 | 72.8 |  |  |  |  |  |  |
| **Asthma** | No | 5127 (85.64) | 4621 | 90.1 | - | - | - |  |  |  |
|  | Yes | 77 (1.29) | 70 | 90.9 | 1.01 | [0.95 ; 1.07] | 0.79 |  |  |  |
|  | Missing Asthma | 783 (13.08) | 571 | 72.9 |  |  |  |  |  |  |
