# Supplementary material for: First wave COVID-19 pandemic in Senegal: Epidemiological and clinical characteristics
Source: PLoS One. 2022 Sep 20;17(9):e0274783. doi: 10.1371/journal.pone.0274783 (PMC9488827; doi:10.1371/journal.pone.0274783)
Supplement: S5 Table — (DOCX) [file pone.0274783.s007.docx]

**Table S5.** Estimates of epidemiological parameters of COVID-19 outbreak in Senegal and the 3 most affected regions

|  | Dakar | Diourbel | Ziguinchor | SENEGAL |
| --- | --- | --- | --- | --- |
| Reproduction number  mean [95% CI] | 1.171 [1.17 ; 1.173] | 1.117 [1.113 ; 1.122] | 1.056 [1.054 ; 1.059] | 1.161 [1.159 ; 1.162] |
| Growth rate  mean [95% CI] | 0.034 [0.03 ; 0.037] | 0.023 [0.015 ; 0.030] | 0.011 [0.007 ; 0.016] | 0.031 [0.028 ; 0.034] |
| Doubling time  number of days [95% CI] | 20 [19 ; 23] | 30 [23 ; 46] | 63 [43 ; 99] | 22 [20 ; 25] |
