# Supplementary material for: First wave COVID-19 pandemic in Senegal: Epidemiological and clinical characteristics
Source: PLoS One. 2022 Sep 20;17(9):e0274783. doi: 10.1371/journal.pone.0274783 (PMC9488827; doi:10.1371/journal.pone.0274783)
Supplement: S6 Table — (DOCX) [file pone.0274783.s008.docx]

**Table S6.** COVID-19 Attack rate per 100,000 inhabitants(hbts) by gender and health district

| **Region** | **District name** | **Number of confirmed cases** | | | **Population size** | | | **Attack rate ( for 100,000 hbts)** | | |
| --- | --- | --- | --- | --- | --- | --- | --- | --- | --- | --- |
|  |  | **Male** | **Female** | **Total** | **Male** | **Female** | **Total** | **Male** | **Female** | **Total** |
| DAKAR | Dakar Sud | 1276 | 949 | 2225 | 111363 | 97237 | 208601 | 1145.80 | 975.97 | 1066.63 |
|  | Dakar Centre | 1073 | 1030 | 2103 | 200553 | 197431 | 397986 | 535.02 | 521.70 | 528.41 |
|  | Dakar Ouest | 1108 | 1048 | 2156 | 125398 | 132921 | 258319 | 883.59 | 788.44 | 834.63 |
|  | Dakar Nord | 722 | 610 | 1332 | 269250 | 266821 | 536070 | 268.15 | 228.62 | 248.48 |
|  | Guediawaye | 444 | 372 | 816 | 199745 | 203241 | 402987 | 222.28 | 183.03 | 202.49 |
|  | Yeumbeul | 79 | 49 | 128 | 164414 | 159940 | 324354 | 48.05 | 30.64 | 39.46 |
|  | Keur Massar | 138 | 101 | 239 | 143007 | 142778 | 285785 | 96.50 | 70.74 | 83.63 |
|  | Pikine | 98 | 79 | 177 | 203715 | 198066 | 401782 | 48.11 | 39.89 | 44.05 |
|  | Mbao | 282 | 222 | 504 | 212332 | 206961 | 419296 | 132.81 | 107.27 | 120.20 |
|  | Rufisque | 79 | 159 | 238 | 134728 | 135511 | 270238 | 58.64 | 117.33 | 88.07 |
|  | Diamniadio | 137 | 83 | 220 | 79759 | 79227 | 158986 | 171.77 | 104.76 | 138.38 |
|  | Sangalkam | 92 | 106 | 198 | 85982 | 84634 | 170617 | 107.00 | 125.25 | 116.05 |
| THIES | Joal-Fadhiouth | 0 | 0 | 0 | 50932 | 46256 | 97188 | 0.00 | 0.00 | 0.00 |
|  | Mbour | 7 | 10 | 17 | 223234 | 215360 | 438595 | 3.14 | 4.64 | 3.88 |
|  | Thiadiaye | 0 | 0 | 0 | 92176 | 92842 | 185019 | 0.00 | 0.00 | 0.00 |
|  | Popenguine | 3 | 0 | 3 | 43375 | 44534 | 87910 | 6.92 | 0.00 | 3.41 |
|  | Thies | 4 | 12 | 16 | 244212 | 249101 | 493313 | 1.64 | 4.82 | 3.24 |
|  | Khombole | 0 | 0 | 0 | 83911 | 84540 | 168449 | 0.00 | 0.00 | 0.00 |
|  | Pout | 0 | 0 | 0 | 72576 | 73084 | 145661 | 0.00 | 0.00 | 0.00 |
|  | Tivaouane | 0 | 0 | 0 | 177765 | 177728 | 355489 | 0.00 | 0.00 | 0.00 |
|  | Mekhe | 0 | 0 | 0 | 95825 | 95385 | 191207 | 0.00 | 0.00 | 0.00 |
| DIOURBEL | Bambey | 11 | 1 | 12 | 180743 | 191141 | 371884 | 6.09 | 0.52 | 3.23 |
|  | Diourbel | 26 | 21 | 47 | 163993 | 169071 | 333060 | 15.85 | 12.42 | 14.11 |
|  | Mbacké | 16 | 20 | 36 | 91395 | 94901 | 186297 | 17.51 | 21.07 | 19.32 |
|  | Touba | 322 | 218 | 540 | 453555 | 514707 | 968261 | 70.99 | 42.35 | 55.77 |
| FATICK | Fatick | 48 | 19 | 67 | 115540 | 118419 | 233965 | 41.54 | 16.04 | 28.64 |
|  | Dioffior | 2 | 0 | 2 | 48589 | 47124 | 95712 | 4.12 | 0.00 | 2.09 |
|  | Niakhar | 3 | 0 | 3 | 47829 | 50250 | 98078 | 6.27 | 0.00 | 3.06 |
|  | Passy | 4 | 3 | 7 | 34266 | 35921 | 70187 | 11.67 | 8.35 | 9.97 |
|  | Foundiougne | 0 | 0 | 0 | 31152 | 32771 | 63925 | 0.00 | 0.00 | 0.00 |
|  | Sokone | 6 | 3 | 9 | 107698 | 110539 | 218236 | 5.57 | 2.71 | 4.12 |
|  | Gossas | 1 | 1 | 2 | 60931 | 59758 | 120688 | 1.64 | 1.67 | 1.66 |
| KAFFRINE | Kaffrine | 5 | 3 | 8 | 130320 | 136677 | 266999 | 3.84 | 2.19 | 3.00 |
|  | Birkilane | 2 | 0 | 2 | 64022 | 66105 | 130126 | 3.12 | 0.00 | 1.54 |
|  | Koungheul | 4 | 2 | 6 | 106389 | 103735 | 210120 | 3.76 | 1.93 | 2.86 |
|  | Malem Hoddar | 0 | 0 | 0 | 61940 | 59760 | 121703 | 0.00 | 0.00 | 0.00 |
| KAOLACK | Kaolack | 101 | 58 | 159 | 188941 | 199347 | 388285 | 53.46 | 29.09 | 40.95 |
|  | Ndoffane | 1 | 1 | 2 | 109050 | 108773 | 217822 | 0.92 | 0.92 | 0.92 |
|  | Nioro du Rip | 19 | 6 | 25 | 218434 | 224186 | 442623 | 8.70 | 2.68 | 5.65 |
|  | Guinguineo | 5 | 0 | 5 | 71877 | 70958 | 142837 | 6.96 | 0.00 | 3.50 |
| KEDOUGOU | Kedougou | 63 | 44 | 107 | 50921 | 47914 | 98835 | 123.72 | 91.83 | 108.26 |
|  | Salemata | 1 | 0 | 1 | 13750 | 14082 | 27830 | 7.27 | 0.00 | 3.59 |
|  | Saraya | 45 | 18 | 63 | 34598 | 29247 | 63847 | 130.07 | 61.54 | 98.67 |
| KOLDA | Kolda | 64 | 57 | 121 | 154051 | 151184 | 305233 | 41.54 | 37.70 | 39.64 |
|  | Velingara | 35 | 36 | 71 | 174653 | 170772 | 345427 | 20.04 | 21.08 | 20.55 |
|  | Medina Yoro Foulah | 0 | 0 | 0 | 87003 | 84339 | 171340 | 0.00 | 0.00 | 0.00 |
|  | Kebemer | 1 | 2 | 3 | 105442 | 108543 | 213989 | 0.95 | 1.84 | 1.40 |
|  | Darou Mousty | 0 | 1 | 1 | 51262 | 49379 | 100645 | 0.00 | 2.03 | 0.99 |
|  | Linguere | 4 | 3 | 7 | 67247 | 66439 | 133685 | 5.95 | 4.52 | 5.24 |
|  | Dahra | 0 | 0 | 0 | 80737 | 79332 | 160072 | 0.00 | 0.00 | 0.00 |
|  | Coki | 2 | 1 | 3 | 40140 | 36907 | 77047 | 4.98 | 2.71 | 3.89 |
|  | Keur Momar Sarr | 0 | 0 | 0 | 44712 | 47158 | 91870 | 0.00 | 0.00 | 0.00 |
|  | Louga | 23 | 14 | 37 | 95208 | 102026 | 197233 | 24.16 | 13.72 | 18.76 |
|  | Sakal | 0 | 1 | 1 | 41948 | 45121 | 87070 | 0.00 | 2.22 | 1.15 |
| MATAM | Matam | 26 | 6 | 32 | 124 445 | 127 725 | 252 168 | 20.89 | 4.70 | 12.69 |
|  | Thilogne | 2 | 0 | 2 | 50 311 | 52 685 | 102 996 | 3.98 | 0.00 | 1.94 |
|  | Kanel | 2 | 2 | 4 | 151 434 | 159 418 | 310 850 | 1.32 | 1.25 | 1.29 |
|  | Ranerou-Ferlo | 1 | 0 | 1 | 34 005 | 32 844 | 66 848 | 2.94 | 0.00 | 1.50 |
| SAINT-LOUIS | Dagana | 1 | 1 | 2 | 47967 | 46803 | 94772 | 2.08 | 2.14 | 2.11 |
|  | richard Toll | 38 | 32 | 70 | 101897 | 93635 | 195531 | 37.29 | 34.18 | 35.80 |
|  | Podor | 0 | 3 | 3 | 120954 | 126938 | 247891 | 0.00 | 2.36 | 1.21 |
|  | Pete | 0 | 0 | 0 | 96418 | 101004 | 197423 | 0.00 | 0.00 | 0.00 |
|  | Saint Louis | 104 | 69 | 173 | 177244 | 178879 | 356124 | 58.68 | 38.57 | 48.58 |
| SEDHIOU | Sédhiou | 35 | 63 | 98 | 97217 | 93905 | 191118 | 36.00 | 67.09 | 51.28 |
|  | Boukiling | 20 | 5 | 25 | 93645 | 90199 | 183842 | 21.36 | 5.54 | 13.60 |
|  | Goudomp | 0 | 0 | 0 | 98941 | 98200 | 197138 | 0.00 | 0.00 | 0.00 |
| TAMBACOUNDA | Bakel | 0 | 0 | 0 | 54699 | 53861 | 108561 | 0.00 | 0.00 | 0.00 |
|  | Kidira | 0 | 0 | 0 | 35268 | 33937 | 69208 | 0.00 | 0.00 | 0.00 |
|  | Tambacounda | 26 | 16 | 42 | 148815 | 146204 | 295018 | 17.47 | 10.94 | 14.24 |
|  | Makacolibantang | 0 | 1 | 1 | 44488 | 43456 | 87943 | 0.00 | 2.30 | 1.14 |
|  | Goudiry | 37 | 35 | 72 | 47164 | 46615 | 93780 | 78.45 | 75.08 | 76.78 |
|  | Dianke makha | 0 | 0 | 0 | 26062 | 27174 | 53236 | 0.00 | 0.00 | 0.00 |
|  | Koumpentoum | 0 | 0 | 0 | 84470 | 79938 | 164406 | 0.00 | 0.00 | 0.00 |
| ZIGUINCHOR | Bignona | 66 | 38 | 104 | 84268 | 79411 | 163680 | 78.32 | 47.85 | 63.54 |
|  | Thionk-Essyl | 7 | 5 | 12 | 25840 | 25068 | 50909 | 27.09 | 19.95 | 23.57 |
|  | Diouloulou | 4 | 1 | 5 | 52899 | 47064 | 99963 | 7.56 | 2.12 | 5.00 |
|  | Oussouye | 14 | 7 | 21 | 31074 | 29121 | 60194 | 45.05 | 24.04 | 34.89 |
|  | Ziguinchor | 217 | 201 | 418 | 156910 | 152296 | 309206 | 138.30 | 131.98 | 135.18 |
