# Supplementary material for: First wave COVID-19 pandemic in Senegal: Epidemiological and clinical characteristics
Source: PLoS One. 2022 Sep 20;17(9):e0274783. doi: 10.1371/journal.pone.0274783 (PMC9488827; doi:10.1371/journal.pone.0274783)
Supplement: S1 File — (DOCX) [file pone.0274783.s009.docx]

**Senegalese District Medical Officers (MCDs)**

| **Region** | **Health District** | **Name** | **E-mail** |
| --- | --- | --- | --- |
| Dakar | Dakar Centre (Gaspard Kamara) | Dr Mbengue | modouthioro@gmail.com |
| Dakar | Dakar Nord (Nabil Choucaire) | Dr Daba SY DIAO | dabasydiao@yahoo.fr |
| Dakar | Dakar Ouest (Philippe Maguilène Senghor) | Dr Abdou Karim DIOP | akdnse@gmail.com |
| Dakar | Dakar Sud (Institut d’Hygiène Sociale) | Dr Maty DIOUF | sakhomaty@gmail.com |
| Dakar | Diamniado | Dr Bouna SALL | bouna.sall1@gmail.com |
| Dakar | Guédiawaye (Roi Baudoin) | Dr Guindho DIOP | ghindo@live.fr |
| Dakar | Keur Massar | Dr Bouna NDIAYE | mbounandiaye@hotmail.fr |
| Dakar | Mbao (Khadimou Rassoul) | Dr Diambogne NDOUR | csantembao@sentoo.sn |
| Dakar | Pikine (Dominique) | Dr Assane NDIAYE | ndiayassane1@yahoo.fr |
| Dakar | Rufisque (Youssou Mbargane Diop) | Dr Mbaye THIAM | mthiam20052000@yahoo.fr |
| Dakar | Sangalkam | Dr DIEDHIOU | bocarbayla@gmail.com |
| Dakar | Yeumbeul | Dr Pape Samba DIEYE | psdieye@yahoo.fr |
| Diourbel | Bambeye | Dr Standeur Nabi KALY | standeurnabikaly@gmail.com |
| Diourbel | Diourbel | Dr Moussa NDIAYE | tonsndiaye@hotmail.fr |
| Diourbel | Mbacké | Dr Adama AIDARA MBACKE | dockarara1@yahoo.fr |
| Diourbel | Touba | Dr Ndeye Maguette DIOP | cstouba@yahoo.fr |
| Fatick | Dioffior | Dr Mamadou NDIAYE | districtdiofior@yahoo.fr |
| Fatick | Fatick | Dr Abiboulaye SALL | docteurabibsall@gmail.com |
| Fatick | Foundiougne | Dr Faly DIOP NDIAYE | ndiayefaly@yahoo.fr |
| Fatick | Gossas | Dr Amady BA | bamady1@yahoo.fr |
| Fatick | Niakhar | Dr Felix DIOUF | diouffelix@gmail.com |
| Fatick | Passy | Dr Mbaye THIOYE | mbayethioye72@gmail.com |
| Fatick | Sokone | Dr Ahmadou Bouya NDAO | bouyandao2017@gmail.com |
| Kaffrine | Birkilane | Dr Papa Birame SECK | seckpapabirahim@yahoo.fr |
| Kaffrine | Kaffrine | Dr Ndèye Mbacké KANE | mbackekane2007@yahoo.fr |
| Kaffrine | Koungheul | DR El Hadji Malick NIANG | drelmalickniang@gmail.com |
| Kaffrine | Malem Hodar | Dr Ibrahima DIALLO | dialloocci@yahoo.fr |
| Kaolack | Guinguinéo | Dr Amadou Mbaye DIOUF | ambdiouf@yahoo.fr |
| Kaolack | Kaolack | Dr Niène SECK | nieneseck@gmail.com |
| Kaolack | Ndoffane | Dr Demba War SALL DIENG | dembawar75@yahoo.fr |
| Kaolack | Nioro | Dr Aboubakry KABA | aboukaba78@yahoo.fr |
| Kedougou | Kédougou | Dr Fodé DANFAKHA (MCA) | deffode47@gmail.com |
| Kedougou | Salémata | Dr DIENE | dienedjibril@yahoo.fr |
| Kedougou | Saraya | Dr Evrard Jocelyn Désiré KABOU | evjodeka@yahoo.fr |
| Kolda | Kolda | Dr Souleymane SAGNA | sagna81souleymane@yahoo.fr |
| Kolda | Médina Yoro Foulah | Dr Boubacar KANDE | drkande2011@yahoo.fr |
| Kolda | Vélingara | Dr Omar SANE | omarsane77@gmail.com |
| Louga | Dahra | Dr Adou NDIAYE | modouthioro@gmail.com |
| Louga | Darou Mousty | Dr Mamadou NDIAYE | drndiaye@yahoo.fr |
| Louga | Kébémer | Dr Ababacar MBAYE | khalifaababacar123@yahoo.fr |
| Louga | Keur Momar Sarr | Dr Babacar SALL | sallbabs@yahoo.fr |
| Louga | Koki | Dr El Hadj Malick DIOUF | elhadjimalickdiouf20@yahoo.fr |
| Louga | Linguère | Dr Pape Saliou NDOYE | bayezale@hotmail.com |
| Louga | Louga | Dr Kalidou BA | kalsdouba@gmail.com |
| Louga | Sakal | Mame Late MBENGUE | mamelate@live.fr |
| Matam | Kanel | Dr Khalifa Ababacar FALL | fallkhalifaababacar@hotmail.com |
| Matam | Matam | Dr Latyr DIOUF | latyrb@yahoo.fr |
| Matam | Ranérou | Dr Aliou NDOUR | alguereo@yahoo.fr |
| Matam | Thilogne | DR Mamadou Sarifou BA | sarifouba10@yahoo.fr |
| Saint-Louis | Dagana | Dr Hamidou DIALLO | midzodia77@hotmail.fr |
| Saint-Louis | Pété | Dr Mamadou NDIAYE | mbolle9@yahoo.fr |
| Saint-Louis | Podor | Dr Malick Hanne | nawndu.e@gmail.com |
| Saint-Louis | Richard Toll | Dr Coumba Ndoffene DIOUF | cndiouf@yahoo.fr |
| Saint-Louis | Saint-Louis | Dr SERIGNE AMDY THIAM | serignamdy@gmail.com |
| Sedhiou | Bounkiling | Dr Bou DIARRA | arraiduab@yahoo.fr |
| Sedhiou | Goudomp | Dr Christophe Koidi KANFOM | drkanfom2012@gmail.com |
| Sedhiou | Sédhiou | Dr Diabéle DRAME | diabeledrame@yahoo.fr |
| Tambacounda | Bakel | Dr Doudou DIALLO | dialdoudou1@yahoo.fr |
| Tambacounda | Diankhé makhan | Dr Tahirou MBAYE | mbayetairou76@hotmail.fr |
| Tambacounda | Goudiry | Faliliou GUEYE | faliliougueye@yahoo.fr |
| Tambacounda | Kidira | Dr Dame | damnd@hotmail.fr |
| Tambacounda | Koumpentoum | Dr El Hadji Malick Abdoulaye DIOP | docdiop82@gmail.com |
| Tambacounda | Makacoulibantang | Dr Alseyni DIALLO | docalseyni2@gmail.com |
| Tambacounda | Tambacounda | Dr Tidiane GADIAGA | tidianegadiaga@yahoo.fr |
| Thies | Joal Fadiouth | Dr Ndèytou DIAGNE SEYE | mamndey@yahoo.fr |
| Thies | Khombole | Dr El Hadji DOUCOURE | ladji79@gmail.com |
| Thies | Mbour | Dr Fatma FALL | fsfall@hotmail.fr |
| Thies | Mékhé | Dr Ndeye Amy BA | ndeyeamyba@yahoo.fr |
| Thies | Popenguine | Dr Youssouph TINE | youtine@gmail.com |
| Thies | Pout | Dr Malick BADIANE | serignemalick@yahoo.fr |
| Thies | Thiadiaye | Dr Youssou MBAYE | youmbaye9@yahoo.fr |
| Thies | Thiès | Dr Moustapha M. FAYE | moustapha75@gmail.com |
| Thies | Tivaouane | Dr Pape Ibrahima CAMARA | pikamara@yahoo.fr |
| Ziguinchor | Bignona | Mamadou Lamine SAGNA | mlsagna@yahoo.fr |
| Ziguinchor | Diouloulou | Dr Mahmadou NDIAYE | mahmadou2002@yahoo.fr |
| Ziguinchor | Oussouye | Dr Gabriel Massène SENGHOR | gabimassene@gmail.com |
| Ziguinchor | Thionck-Essyl | Abdel Kader Souandy SARR | souandysarr@yahoo.fr |
| Ziguinchor | Ziguinchor | Dr Jean Jacques MALOMAR | jjmalomar@gmail.com |
