# Supplementary figures and images for: First wave COVID-19 pandemic in Senegal: Epidemiological and clinical characteristics
Source: PLoS One. 2022 Sep 20;17(9):e0274783. doi: 10.1371/journal.pone.0274783 (PMC9488827; doi:10.1371/journal.pone.0274783)

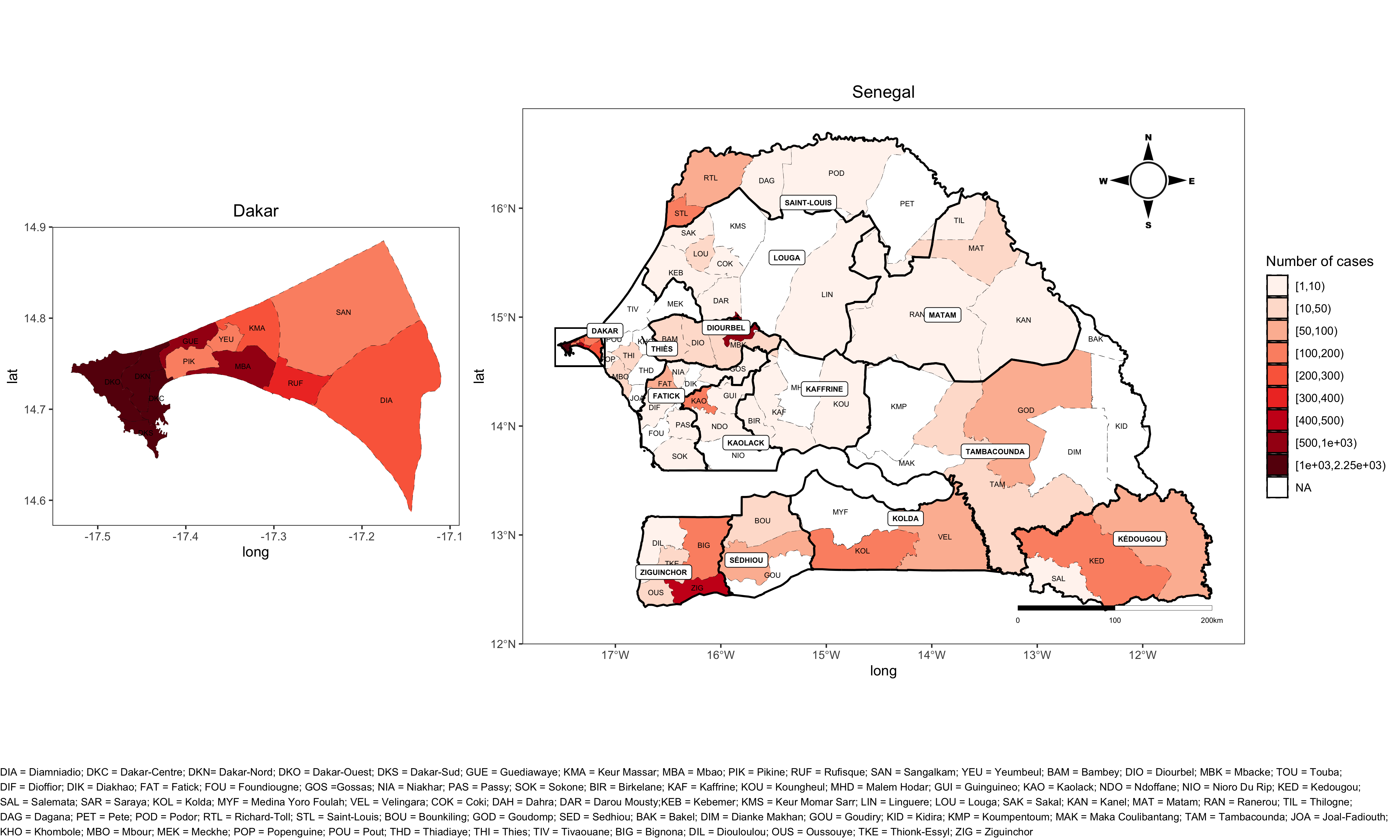

Supplement: S2 File — (ZIP) [file pone.0274783.s010.zip › Map_Creating/Figure4.tiff]
